# Impact of N-Acetylcysteine on the Gut Microbiota in the Piglets Infected With Porcine Epidemic Diarrhea Virus

**DOI:** 10.3389/fvets.2020.582338

**Published:** 2021-01-12

**Authors:** Tao Wu, Yang Lyu, Xueni Li, Mengjun Wu, Kui Yu, Siyuan Li, Changzheng Ji, Qian Zhang, Yanyan Zhang, Di Zhao, Dan Yi, Yongqing Hou

**Affiliations:** Hubei Key Laboratory of Animal Nutrition and Feed Science, Wuhan Polytechnic University (WPHU), Wuhan, China

**Keywords:** N-acetylcysteine, PEDV, dysbiosis, microbiome, piglets

## Abstract

This study was to investigate the impact of N-acetylcysteine (NAC) on the gut microbiota in the healthy piglets and the piglets infected with porcine epidemic diarrhea virus (PEDV). Forty seven-day-old piglets were allocated into four groups: control group, NAC group (supplemented with 50 mg/kg body weight NAC), PEDV group (inoculated with 10^4.5^ TCID_50_ PEDV), and PEDV+NAC group (PEDV infection + NAC supplementation). The intestinal content was collected for DNA extraction and Illumina sequencing. The PEDV-infected piglets displayed distinct bacterial communities compared to the healthy piglets. PEDV infection decreased the abundance of *Shigella* and increased the abundance of *Lactobacillus, Odoribacter, Anaerovibrio, Helicobacter*, unclassified *Lachnospiraceae*, and *Sutterella*; affected several functions associated with metabolism, barrier, and immune. NAC supplementation decreased the abundance of unclassified *Rikenellaceae* and increased the abundance of *Lactobacillus, Streptococcus*, and *Enterococcus* in the healthy piglets, decreased the abundance of *Oscillospira* and *Prevotella* and increased the abundance of *Lactobacillus* in the PEDV-infected piglets; altered multiple functions involving in amino acid metabolism, cell signaling, cellular community, disease-related pathways, endocrine, and excretory system. In conclusion, PEDV infection caused severe dysbiosis of gut microbiome, whereas NAC supplementation played a positive role in regulating the gut microbiome during PEDV infection. Therefore, substances that can regulate gut microbiota could be ideal candidates to prevent or treat PEDV infection.

## Introduction

Great progress has been made to understand the role of gut microbiota in humans and animals alike (1). Biological functions of the gut microbiota to the host pertain to nutrition (2), metabolism (3), barrier, and immune (4), which are still expanding. Nevertheless, the relationship between gut microbiota and diseases remains unclear (5). For instance, increasing evidence have revealed that gut microbiota plays a vital role in the regulation, elimination, and potentiation of infectious diseases (6), while profound shifts of gut microbiota have frequently been reported in various diseases as well (7). Moreover, several enteric pathogens, such as *Salmonella, Clostridium perfringens*, and *Escherichia coli*, can also be found in healthy mammals (8). Therefore, more experiments are warranted to be established for a deeper understanding of microbiota alterations during diseases.

Porcine epidemic diarrhea virus (PEDV) received increasing attention since the large-scale outbreaks in the last decades (9). PEDV is a devastating cause of diarrhea in pigs, which induces great economic losses to the pig industry worldwide (10). It causes severe diarrhea, vomiting, anorexia, dehydration, weight loss and high morbidity, and mortality in pigs of any age (11). Currently, research on PEDV mainly focused on molecular characterization, evolution, pathology, the host immune response and vaccinations, only limited knowledge has been expanded to the relationship between PEDV infection and porcine microbiota (12). Recent research has reported that PEDV infection induced dysbiosis of fecal and colonic microbiota in piglets and sows, leading to an increase in pathogenic bacteria and a decrease in commensal bacteria (13–17). However, only few studies focused on the microbiota alterations associated with the (expecting) anti-PEDV substances or compounds.

N-acetylcysteine (NAC) is a source of sulfhydryl groups in cells and scavenger of free radicals as it interacts with reactive oxygen species such as hydroxide and hydrogen peroxide (18). NAC is rapidly metabolized by the small intestine to produce glutathione. Enterocytes, colonocytes, and other intestinal cells readily transport NAC and convert it into L-cysteine (19), which helps to maintain the integrity, growth, and function of the intestinal mucosa (20). Moreover, glutathione is a well-described and established antioxidant, it participates in detoxification of xenobiotics, regulation of cellular growth, modulation of immune response, and even inherent antibacterial properties (21), which is an important modulator of antibiotic activity in bacteria (22). This makes NAC a promising supplement for the piglets to regulate the porcine gut microbiome and alleviate dysbiosis during pathogenic infection (23), including PEDV (24). Our previous study also demonstrated that dietary NAC supplementation improved intestinal integrity and functions in PEDV-infected piglets (25). Based on the potential anti-PEDV effect of NAC in the piglets, this study was conducted to evaluate the impact of NAC on small intestinal microbiota in suckling piglets infected with PEDV. Findings of this study can contribute to understand the role of the gut microbiota during PEDV infection and provide vital clues for the treatment for PEDV infection.

## Materials and Methods

### Animals Experiments and Sample Collection

The animal use protocol for this research was approved by the Animal Care and Use Committee of Wuhan Polytechnic University (1204-2016-0028). Forty seven-day-old crossbred (Duroc × Landrace × Large White) healthy piglets with 3.17 ± 0.25 kg initial body weight were purchased from a PEDV-negative farm. All piglets were randomly allocated into one of four treatment groups (Control, NAC, PEDV, PEDV+NAC; 10 replicates per group). All piglets were housed in clean pens with strict control of cross-infection. A commercial milk replacer (Wuhan Anyou Feed Co., Ltd.; Wuhan, China) formulated to meet the nutrition requirement for suckling piglets, was used as the basal diet during the trial. Prior to feeding, the milk replacer (powder) was dissolved in warm water (45–55°C) to form a liquid feed (dry matter content of 20%), as described by Wang et al. (25).

The entire trial period was 12 days. During day 5–9 of the trial, the piglets in the NAC and the PEDV + NAC groups were orally administered with 50 mg/kg body weight NAC (Sigma Chemical Inc., St. Louis, MO, USA; dissolved in the liquid milk replacer), and the piglets in the other two groups received the same volume of the liquid milk replacer. On day 9 of the trial, PEDV at a dose of 10^4.5^ TCID_50_ (50% tissue culture infectious dose) per piglet was orally inoculated to the piglets in the PEDV and the PEDV + NAC groups, the same volume of sterile saline was inoculated to the piglets in control and NAC groups. On day 12, all piglets were sacrificed to obtain the intestinal content from jejunum, ileum, colon, and cecum. All samples were rapidly frozen in liquid nitrogen and then stored at −80°C until analysis.

### DNA Extraction

The bacterial DNA was extracted from the intestinal content using the QIAampDNA Stool Mini Kit (Qiagen, Hilden, Germany) according to the manufacturer's protocol. The extracted DNA was purified by using the QIAamp spin column (Qiagen, Hilden, Germany). Total DNA was quantified by determining the OD 260 nm/OD 280 nm ratio using the NanoDrop®ND-1000A UV-VIS spectrophotometer (Thermo Scientific, Wilmington, DE, USA). The quality of microbial DNA in each sample was evaluated by 1% denatured agarose gel electrophoresis and the quantity of DNA with Picogreen assay. The microbial DNA were normalized with 10 mM Tris buffer (pH 8.5) to 5 ng/ul.

### Amplification and Sequencing

The V3-V4 region of the 16S rRNA gene was amplified by two stage polymerase chain reaction (PCR). The first stage of PCR was processed using the HiFi HotStart ReadyMix kit (Kapa Biosystems, Wilmington, MA, USA) with the forward primer: 5′-TCGTCGGCAGCG TCAGATGTGTATAAGAGACAGCCTACGGGNGGCWGCAG-3′ and the reverse primer 5′-GTCTCGTGGGCTCGGAGATGTGTATAAGAGACAGGACTACHVGGGTATCT AATCC-3′. The total reaction volume of PCR was 25 μL, containing 12.5 μL of 2 × HotStart ReadyMix Buffer, 0.5 μL of each primer (5 mM) and 10 ng of template DNA. PCR thermocycling conditions were denaturation at 95°C for 3 min (1 cycle), 95°C for 30 s, annealing at 55°C for 30 s, elongation at 72°C for 30 s and a final extension at 72°C for 5 min. The second stage was index PCR. This step attaches dual indices and Illumina sequencing adapters using the Nextera XT Index Kit (Illumina, San Diego, CA, USA). The total reaction volume was 50 μL, containing 25 μL of 2 × HotStart ReadyMix Buffer, 5 μL Cleaned PCR product, 5 μL N7 index and 5 μL S5 index primer. PCR thermocycling conditions were 95°C for 3 min (1 cycle), 95°C for 30 s, annealing at 55°C for 30 s, elongation at 72°C for 30 s (8 cycles), and a final extension at 72°C for 5 min. The PCR production was purified with the Agencourt AMPure XP purification system (Beckman, Brea, CA, USA) and the concentration was measured with SpectraMax i3X Multi-mode detection platform (Molecular Devices, San Jose, CA, USA). The purified DNA samples were sequenced using Illumina MiSeq platform (San Diego, CA, USA).

### Data Processing and Statistical Analysis

The raw reads were put for quality control after base calling, and then were subjected to pipeline QIIME for OTU picking and taxonomy as described (26). Alpha diversity (Shannon, Simpson, and Chao1) were calculated, and the one-way analysis of variance (ANOVA) with a Turkey's test as *post-hoc* was used to compare between the groups. The beta diversity (Bray-Curtis Index) was calculated using principal coordinate analysis (PCoA), with further comparison of groups by analysis of similarities (ANOSIM). Dendrograms and colinear relation diagrams were generated using Majorbio Cloud Platform (Shanghai Majorbio Bio-pharm Technology, Shanghai, China). Relative abundance of operational taxonomic units (OTUs) were compared by one-way analysis of variance (ANOVA) using Majorbio Cloud Platform, with a Turkey's test as *post-hoc* and *p*-values adjusted by false discovery rate (FDR). Functional potentials of OTUs were evaluated by KEGG (Kyoto Encyclopedia of Genes and Genomes) pathway enrichment using PICRUSt (http://huttenhower.sph.harvard.edu/galaxy/), a heatmap was generated using TBtools (https://github.com/CJ-Chen/TBtools), and the comparison was processed by ANOVA using Majorbio Cloud Platform with a Turkey's test as *post-hoc*. Significance was set at *p* < 0.05 for all comparisons.

## Results

### Sequencing Data and OUT Clustering

A total of 3,629,889 reads were obtained with an average length of 460.36 bp across all samples, with 3,629,889 reads from jejunum, 4,658,260 reads from ileum, 2,987,358 reads from colon, and 2,936,753 reads from cecum. The good coverage was obtained over 99.9% for all samples, reflecting reliable accuracy of the sequencing.

Evenness, diversity, and richness were calculated by Shannon, Simpson, and Chao1 indices, respectively (Table 1). Compared to the control group, the NAC group increased the Chao1 index in the ileum, the PEDV group increased the Shannon index in the jejunum, and the PEDV and the PEDV+NAC group increased the Simpson index in the jejunum (*p* < 0.05). No significant difference was observed in the colon and cecum.

**Table 1:** Alpha diversity of gut microbiota in piglets.

**Item**	**Control**	**NAC**	**PEDV**	**PEDV+NAC**	***p*-value**
**Jejunum**					
Shannon	1.76 ± 0.66^b^	1.76 ± 0.74^b^	2.32 ± 0.28^ab^	2.72 ± 0.78^a^	0.008
Simpson	0.60 ± 0.21^b^	0.59 ± 0.24^b^	0.80 ± 0.04^a^	0.83 ± 0.14^a^	0.006
Chao1	91.98 ± 5.65	96.19 ± 2.79	94.74 ± 2.76	92.22 ± 4.77	0.134
**Ileum**					
Shannon	1.80 ± 0.74	1.98 ± 0.57	2.11 ± 0.72	2.30 ± 0.58	0.403
Simpson	0.56 ± 0.22	0.63 ± 0.17	0.65 ± 0.21	0.73 ± 0.16	0.329
Chao1	102.57 ± 4.57^b^	106.68 ± 2.04^a^	105.37 ± 4.02^ab^	105.19 ± 4.69^ab^	0.170
**Colon**					
Shannon	3.40 ± 0.19	3.09 ± 0.41	3.34 ± 0.37	3.18 ± 0.42	0.257
Simpson	0.92 ± 0.02	0.89 ± 0.04	0.92 ± 0.03	0.91 ± 0.04	0.207
Chao1	166.09 ± 10.57	159.63 ± 16.28	156.78 ± 25.73	157.53 ± 26.75	0.777
**Cecum**					
Shannon	3.23 ± 0.23	3.10 ± 0.43	3.22 ± 0.40	3.16 ± 0.33	0.850
Simpson	0.90 ± 0.03	0.88 ± 0.05	0.91 ± 0.04	0.91 ± 0.02	0.340
Chao1	162.66 ± 11.78	162.71 ± 12.96	161.02 ± 17.26	148.52 ± 22.34	0.214

a,b *Values with different letters are significantly different (p < 0.05)*.

**Figure 1:**
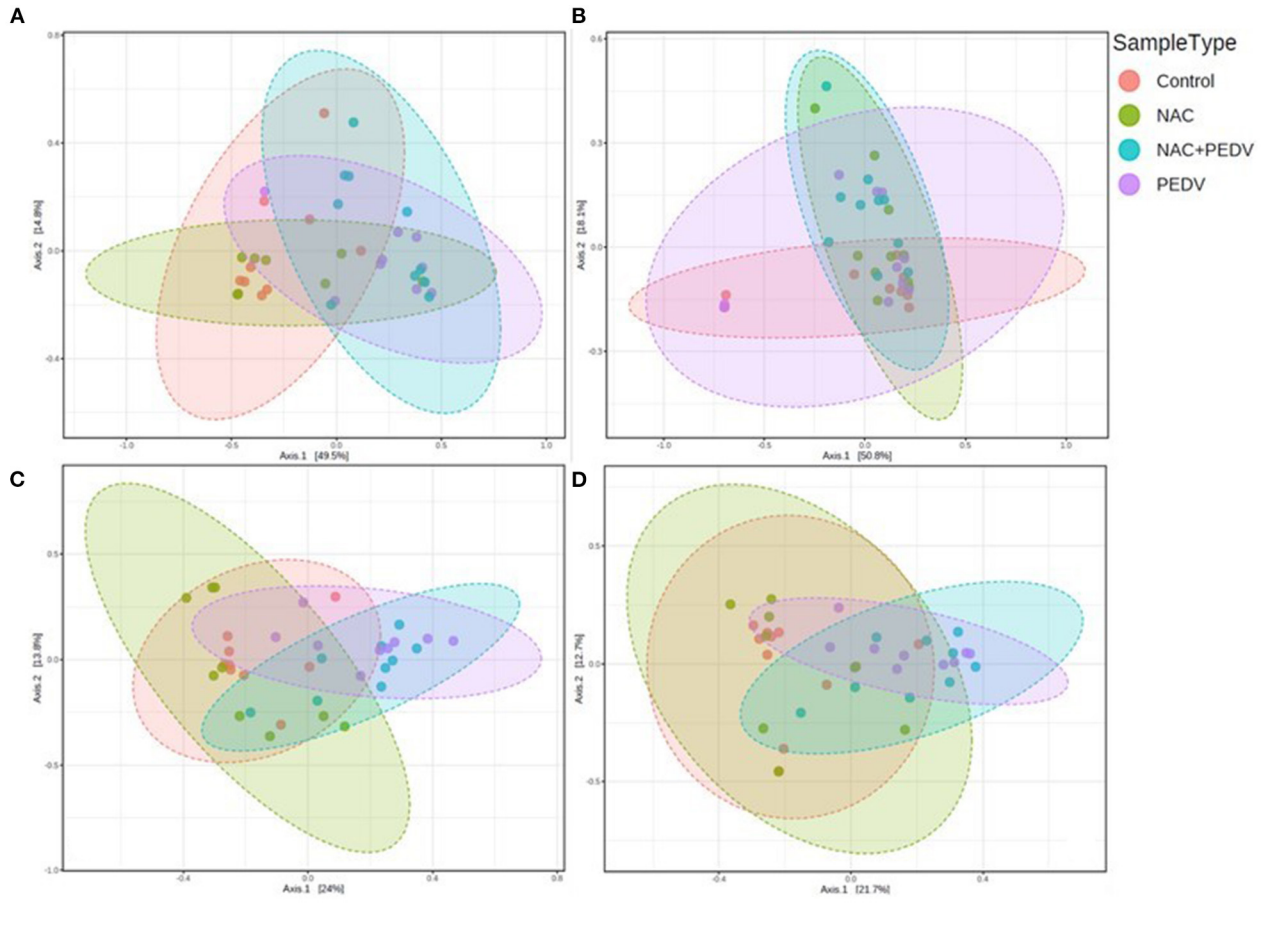
Beta diversity of gut microbiota in piglets. **(A)** Jejunum, *p* = 0.002; **(B)** ileum, *p* = 0.398; **(C)** colon, *p* < 0.001; **(D)** cecum, *p* < 0.001.

Beta diversity was calculated by PCoA based on the Bray-Curtis index. There is no clear clustering in the ileum (*p* = 0.398) (Figure 1B), while distinct bacterial communities were observed in the jejunum (*p* = 0.002) (Figure 1A), colon (*p* < 0.001) (Figure 1C), and cecum (*p* < 0.001) (Figure 1D). Particularly, the result illustrated obvious clusters between the control and the NAC groups as well as between the PEDV and the PEDV+NAC groups in the jejunum, colon, and cecum.

### Structure of Intestinal Microbiota

**Figure 2:**
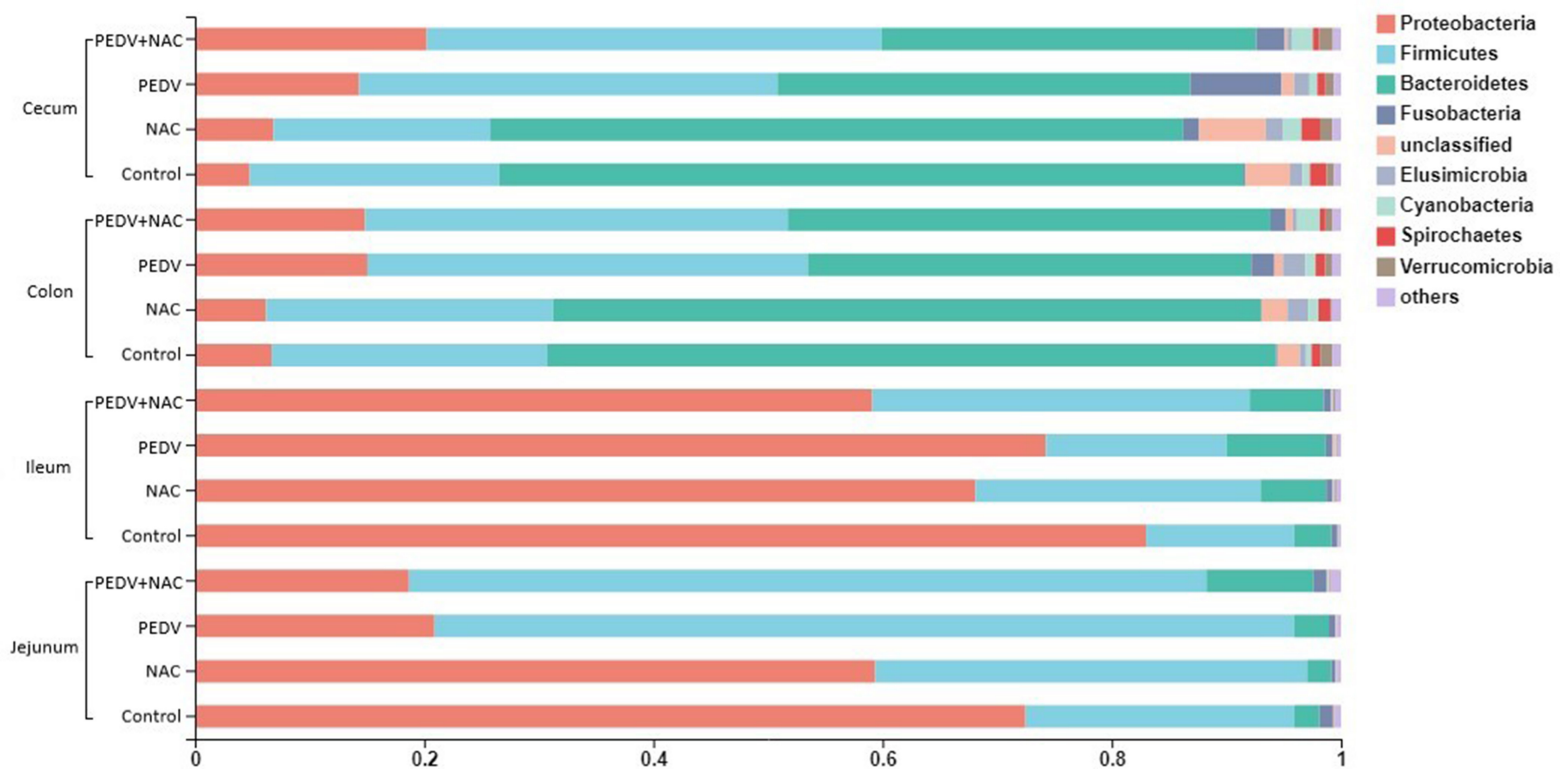
Relative abundance of sequences at the phylum level (%). Only sequences with relative abundance over 0.01% were presented.

The majority of microbiota in all samples was composed of four predominant bacterial phyla: *Proteobacteria, Firmicutes, Bacteroidetes*, and *Fusobacteria*, accounting for an average of over 96% of total sequences (Figure 2). In the jejunum, the proportions of the four phyla in the control group were 72.5, 23.5, 2.2, and 1.2%; that in the NAC group were 59.4, 37.7, 2.1, and 0.4%; that in the PEDV group were 20.9, 75.0, 3.0, and 0.6%; that in the PEDV+NAC group were 18.7, 69.6, 9.4, and 1.2%. In the ileum, the proportions of the four phyla were 83.0, 12.9, 3.3, and 0.5% in the control group; 68.1, 24.9, 5.7, and 0.5% in the NAC group; 74.3, 15.8, 8.6, and 0.6% in the PEDV group; 59.1, 32.9, 6.4, and 0.6% in the PEDV+NAC group. In the colon, the proportions were 6.7, 24.1, 63.6, and 0.1% in the control group; 6.2, 25.0, 61.8, and 0.1% in the NAC group; 15.0, 38.4, 38.7, and 2.0% in the PEDV group, 14.9, 36.9, 42.1, and 1.3% in the PEDV+NAC group. In the cecum, the proportions were 4.8, 21.7, 65.0, and 0.2% in the control group; 6.9, 18.9, 60.4, and 1.4% in the NAC group; 14.3, 36.4, 36.0, and 8.0% in the PEDV group, 20.2, 39.7, 32.7, and 2.5% in the PEDV+NAC group.

**Figure 3:**
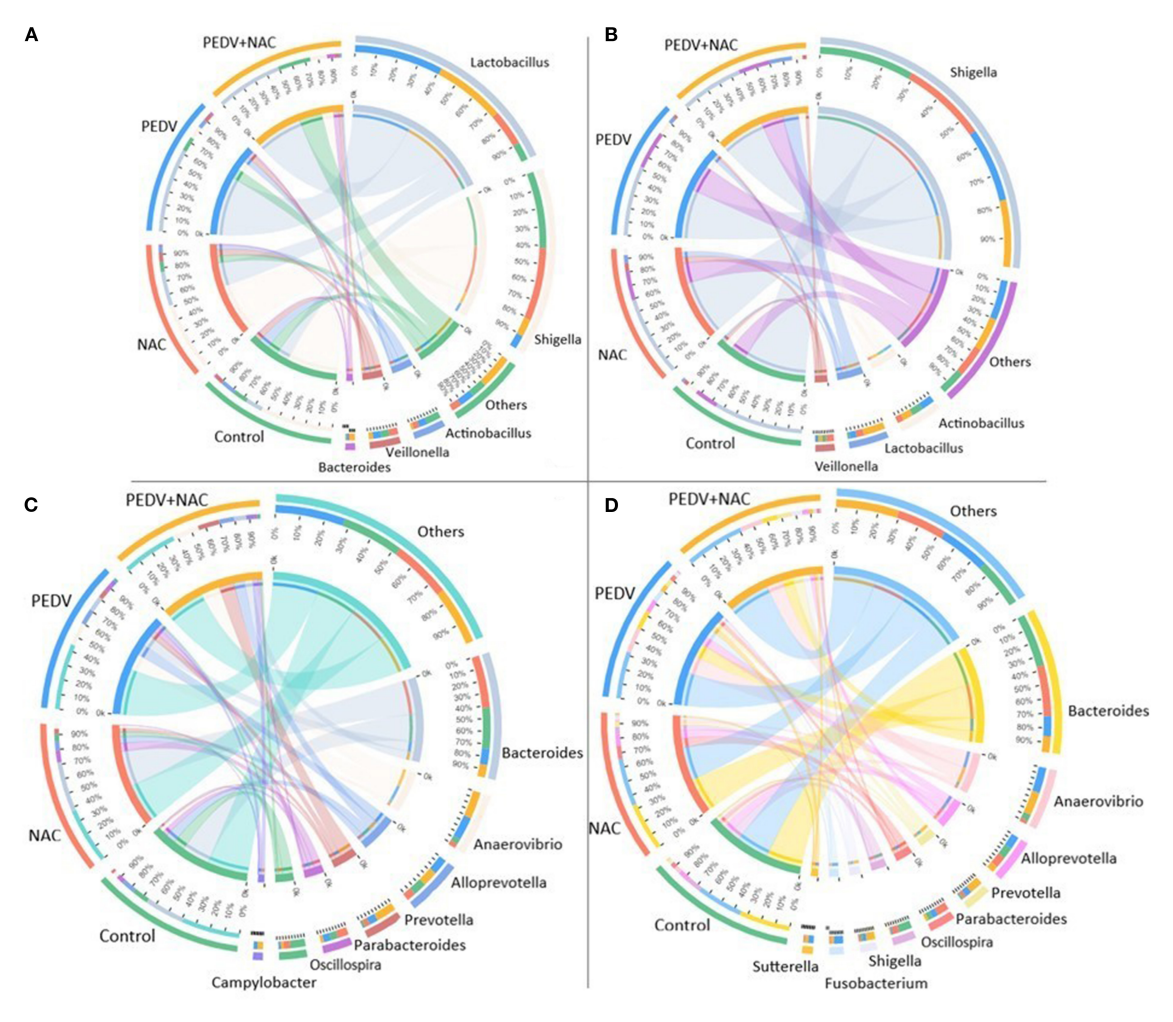
Relative abundance of OTUs at the genus level (%). **(A)** Jejunum; **(B)** ileum; **(C)** colon; **(D)** cecum. Only OTUs with relative abundance over 2% were presented.

A total of 13 genera was identified as the dominant bacterial populations (> 2%) in all groups. *Shigella, Lactobacillus, Actinobacillus* and *Veillonella* were the most abundant genera in the jejunum, accounting for 53, 13, 12, and 6.4% in the control group, 52, 27, 5.1, and 7.1% in the NAC group, 63, 9, 5.7, and 6.1% in the PEDV group, 51, 13, 1.6, and 3.3% in the PEDV+NAC group (Figure 3A). *Shigella, Actinobacillus, Lactobacillus*, and *Veillonella* were the most abundant genera in the ileum, accounting for 63, 7.9, 1.9, and 3.3% in the control group, 59, 5.4, 5.9, and 6.4% in the NAC group, 48, 8.7, 4.4, and 2.4% in the PEDV group, 46, 8.1, 17, and 3.0% in the PEDV+NAC group (Figure 3B). *Bacteroides, Anaerovibrio, Alloprevotella, Prevotella, Parabacteriodies*, and *Oscillospria* were the most abundant genera in the colon, accounting for 27, 3.8, 9.5, 1.9, 5.4, and 11% in the control group, 35, 3.3, 6.4, 3.8, 8.1, and 4.8% in the NAC group, 11, 16, 11, 6.1, 5.3, and 1.7% in the PEDV group, 8.5, 19, 11, 15, 2.3, and 2.2% in the PEDV+NAC group (Figure 3C). *Bacteroides, Anaerovibrio, Alloprevotella, Prevotella, Parabacteriodies*, and *Oscillospria* were the most abundant genera in the cecum, accounting for 36, 4.6, 8.3, 1.7, 3.7, and 10% in the control group, 34, 2.5, 8.3, 2.9, 9.3, and 5% in the NAC group, 14, 18, 9.1, 5, 4.3, and 1.3% in the PEDV group, 11, 16, 4.8, 10, 2.1, and 0.1% in the PEDV+NAC group (Figure 3D).

### Comparison of OTUs Abundance

The differences in the percentage of OTU abundance between all groups were summarized in Table 2. In the jejunum, compared to the control group, both the PEDV and the PEDV+NAC groups decreased the abundance of *Shigella* and *Lachnospiraceae*_g (an unclassified genus that belongs to family *Lachnospiraceae*, same as others), increased the abundance of *Lactobacillus* (*p* < 0.05). No significant difference was observed between the control and the NAC groups as well as between the PEDV and the PEDV+NAC groups. In the ileum, compared to the control group, the NAC group increased the abundance of *Lactobacillus, Streptococcus* and *Enterococcus*, the PEDV group increased the abundance of *Prevotella* and *Odoribacter*, and the PEDV+NAC group increased the abundance of *Lactobacillus* (*p* < 0.05); compared to the PEDV group, the PEDV+NAC group decreased the abundance of *Prevotella* and increased the abundance of *Lactobacillus* (*p* < 0.05). In the colon, compared to the control group, the NAC group decreased the abundance of *Rikenellaceae*_g and increased the abundance of *Acidaminococcus*, the PEDV group decreased the abundance of *Rikenellaceae*_g and increased the abundance of *Anaerovibrio, Lachnospiraceae*_g, *Lachnoclostridium, Helicobacter*, and *Sutterella* (*p* < 0.05); compared to the PEDV group, the PEDV+NAC group increased the abundance of *Allisonella* (*p* < 0.05). In the cecum, compared to the control group, the PEDV group decreased the abundance of *Prevotella* and increased the abundance of *Anaerovibrio, Oscillospira, Lachnospiraceae*_g, *Helicobacter, Sutterella*, and *Butyricimonas* (*p* < 0.05); compared to the PEDV group, the PEDV+NAC group decreased the abundance of *Oscillospira* (*p* < 0.05).

**Table 2:** Comparison of OUT abundance (%).

**Taxonomy**	**Control**	**NAC**	**PEDV**	**PEDV+NAC**	***p***
**Jejunum**					
o_*Enterobacteriales*; f_*Enterobacteriaceae*; g_*Shigella*	36.43 ± 22.29^a^	36.93 ± 20.91^a^	7.44 ± 9.79^b^	7.57 ± 9.23^b^	0.038
o_*Enterobacteriales*; f_*Enterobacteriaceae*; g_*Shigella*	14.94 ± 9.25^a^	16.70 ± 9.91^a^	3.71 ± 4.71^b^	3.18 ± 3.99^b^	0.038
o_*Lactobacillales*; f_*Lactobacillaceae*; g_*Lactobacillus*	0.10 ± 0.09^b^	0.90 ± 1.42^b^	5.31 ± 3.27^a^	4.48 ± 5.29^a^	0.067
o_*Clostridiales*; f_*Lachnospiraceae*; g_	0.33 ± 0.42^a^	0.11 ± 0.09^ab^	0.05 ± 0.06^b^	0.01 ± 0.01^b^	0.067
o_*Lactobacillales*; f_*Lactobacillaceae*; g_*Lactobacillus*	0.02 ± 0.03^b^	0.06 ± 0.07^b^	0.14 ± 0.08^a^	0.15 ± 0.12^a^	0.067
**Ileum**					
o_*Lactobacillales*; f_*Lactobacillaceae*; g_*Lactobacillus*	0.40 ± 0.47^c^	2.63 ± 2.53^b^	1.53 ± 1.78^bc^	8.32 ± 9.49^a^	0.045
o_*Lactobacillales*; f_*Streptococcaceae*; g_*Streptococcus*	0.06 ± 0.07^b^	0.15 ± 0.10^a^	0.04 ± 0.02^b^	0.11 ± 0.11^ab^	0.096
o_*Bacteroidales*; f_*Paraprevotellaceae*; g_*Prevotella*	0.02 ± 0.04^b^	0.01 ± 0.03^b^	0.08 ± 0.09^a^	0.01 ± 0.01^b^	0.096
o_*Lactobacillales*; f_*Enterococcaceae*; g_*Enterococcus*	0.01 ± 0.02^b^	0.06 ± 0.07^a^	0.01 ± 0.01^b^	0.01 ± 0.01^b^	0.096
o_*Bacteroidales*; f_*Odoribacteraceae*; g_*Odoribacter*	0.01 ± 0.01^b^	0.02 ± 0.03^b^	0.05 ± 0.05^a^	0.03 ± 0.03^ab^	0.096
**Colon**					
o_*Selenomonadales*; f_*Selenomonadaceae*; g_*Anaerovibrio*	2.75 ± 4.07^b^	1.40 ± 1.98^b^	7.65 ± 5.76^ab^	9.78 ± 6.09^a^	0.034
o_*Selenomonadales*; f_*Selenomonadaceae*; g_*Anaerovibrio*	1.89 ± 2.91^b^	0.83 ± 1.17^b^	5.19 ± 3.72^a^	5.83 ± 3.47^a^	0.034
o_*Selenomonadales*; f_*Selenomonadaceae*; g_*Anaerovibrio*	1.35 ± 2.04^b^	0.62 ± 0.87^b^	3.83 ± 2.82^a^	4.48 ± 2.75^a^	0.083
o_*Lachnospirales*; f_*Lachnospiraceae*; g_	0.45 ± 0.67^b^	0.08 ± 0.08^b^	3.81 ± 3.70^a^	2.05 ± 2.72^ab^	0.083
o_*Lachnospirales*; f_*Lachnospiraceae*; g_*Lachnoclostridium*	0.23 ± 0.22^b^	0.44 ± 0.39^b^	2.08 ± 1.67^a^	1.50 ± 1.17^ab^	0.083
o_*Campylobacterales*; f_*Helicobacteraceae*; g_*Helicobacter*	0.01 ± 0.01^b^	0.04 ± 0.11^b^	1.31 ± 0.95^a^	0.63 ± 1.12^ab^	0.083
o_*Acidaminococcales*; f_*Acidaminococcaceae*; g_*Acidaminococcus*	0.04 ± 0.09^b^	1.22 ± 1.56^a^	0.01 ± 0.02^b^	0.12 ± 0.32^b^	0.167
o_*Burkholderiales*; f_*Sutterellaceae*; g_*Sutterella*	0.15 ± 0.17^b^	0.05 ± 0.06^b^	0.46 ± 0.51^a^	0.31 ± 0.36^a^	0.167
o_*Oscillospirales*; f_*Oscillospiraceae*; g_	0.05 ± 0.11^b^	0.08 ± 0.17^b^	0.32 ± 0.25^ab^	0.55 ± 0.61^a^	0.167
o_*Lactobacillales*; f_*Lactobacillaceae*; g_*Lactobacillus*	0.06 ± 0.09^b^	0.02 ± 0.04^b^	0.15 ± 0.22^ab^	0.69 ± 0.75^a^	0.167
o_*Veillonellales*; f_*Veillonellaceae*; g_*Allisonella*	0.15 ± 0.41^b^	0.06 ± 0.12^b^	0.02 ± 0.03^b^	0.77 ± 1.03^a^	0.167
o_*Lachnospirales*; f_*Lachnospiraceae*; g_*Lachnoclostridium*	0.05 ± 0.05^b^	0.10 ± 0.07^b^	0.51 ± 0.39^a^	0.35 ± 0.26^ab^	0.167
o_*Bacteroidales*; f_*Rikenellaceae*; g_	0.43 ± 0.45^a^	0.12 ± 0.14^b^	0.02 ± 0.02^b^	0.03 ± 0.05^b^	0.167
**Cecum**					
o_*Selenomonadales*; f_*Selenomonadaceae*; g_*Anaerovibrio*	2.88 ± 4.38^b^	3.08 ± 4.57^b^	9.60 ± 7.13^a^	6.78 ± 5.35^ab^	0.070
o_*Selenomonadales*; f_*Selenomonadaceae*; g_*Anaerovibrio*	1.60 ± 3.10^b^	0.85 ± 0.74^b^	6.86 ± 4.17^a^	4.53 ± 2.91^a^	0.070
o_*Clostridiales*; f_*Ruminococcaceae*; g_*Oscillospira*	0.85 ± 0.72^b^	0.70 ± 0.54^b^	2.97 ± 2.79^a^	1.22 ± 1.41^ab^	0.070
o_*Clostridiales*; f_*Lachnospiraceae*; g_	0.19 ± 0.21^b^	0.60 ± 0.51^b^	1.97 ± 1.69^a^	1.55 ± 1.45^a^	0.124
o_*Bacteroidales*; f_*Prevotellaceae*; g_*Prevotella*	1.23 ± 2.35^a^	1.44 ± 1.65^a^	0.02 ± 0.02^b^	0.02 ± 0.01^b^	0.124
o_*Burkholderiales*; f_*Alcaligenaceae*; g_*Sutterella*	0.15 ± 0.26^b^	0.21 ± 0.25^b^	0.97 ± 0.93^a^	0.47 ± 0.56^ab^	0.124
o_*Clostridiales*; f_*Lachnospiraceae*; g_	0.05 ± 0.05^b^	0.13 ± 0.12^ab^	0.30 ± 0.35^a^	0.16 ± 0.12^ab^	0.124
o_*Bacteroidales*; f_*Odoribacteraceae*; g_*Butyricimonas*	0.09 ± 0.10^b^	0.13 ± 0.12^ab^	0.30 ± 0.35^a^	0.16 ± 0.12^ab^	0.124
o_*Clostridiales*; f_*Ruminococcaceae*; g_*Oscillospira*	0.20 ± 0.17^a^	0.12 ± 0.20^ab^	0.25 ± 0.18^a^	0.08 ± 0.05^b^	0.124

a,b,c *Values with different letters are significantly different (p < 0.05)*.

*f_Lachnospiraceae; g_ means an unclassified genus that belongs to family Lachnospiraceae, same as others*.

### Functional Potentials of OUTs

**Figure 4:**
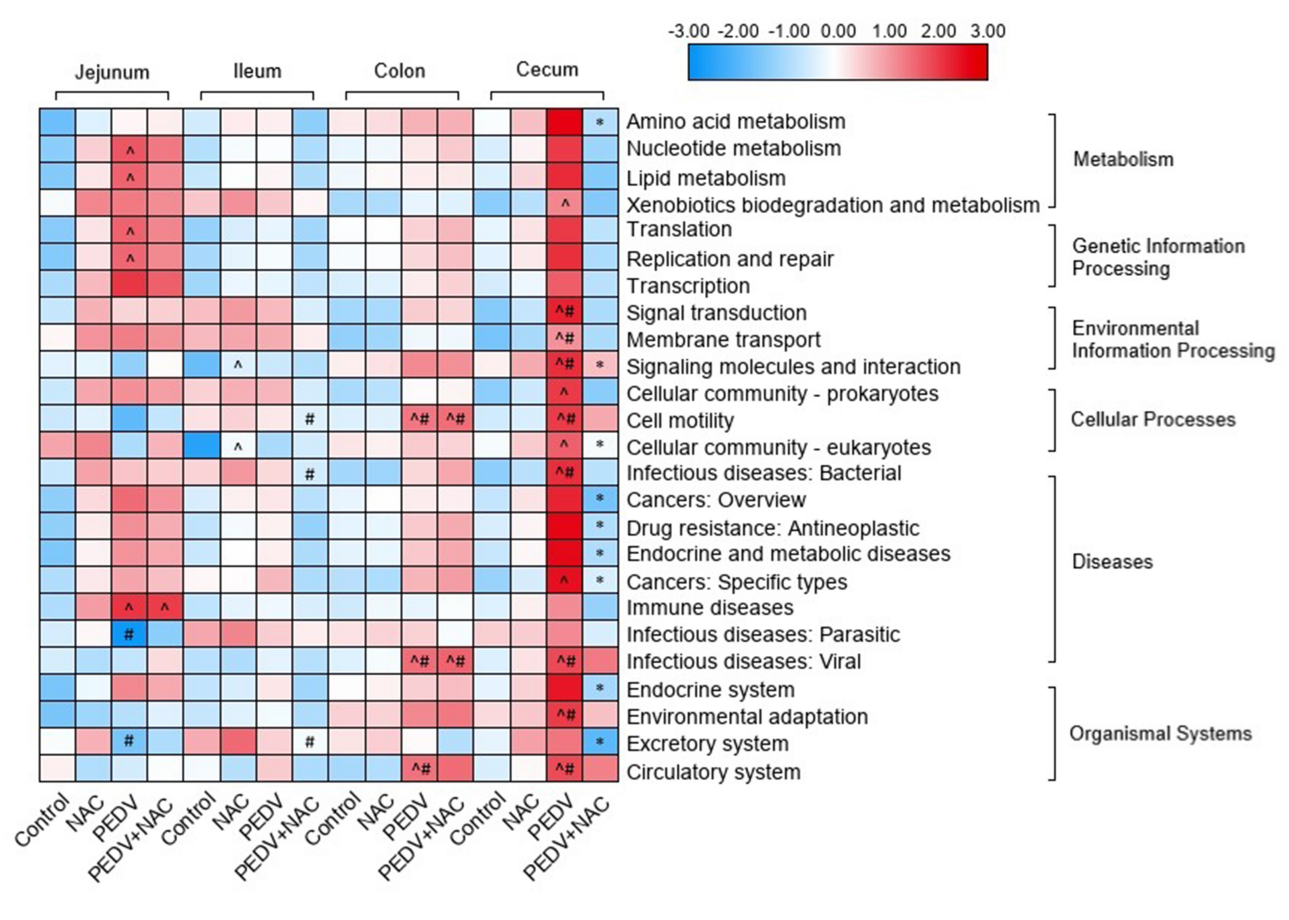
KEGG pathway enrichment of gut microbiome in piglets. Different colors represent the values of log-transformed abundances of KEGG pathways. ^∧^means significant difference compared to the control group (*p* < 0.05); ^#^means significant difference compared to the NAC group (*p* < 0.05); *means significant difference compared to the PEDV group (*p* < 0.05).

Functional potentials of the microbiome profile were evaluated by KEGG pathway enrichment, the relative abundance of 25 pathways was found with significant differences between groups (Figure 4). In the jejunum, compared to the control group, the PEDV group increased the abundance of pathways “nucleotide metabolism,” “lipid metabolism,” “translation,” “replication and repair,” “transcription,” and “immune diseases,” the PEDV+NAC group increased the abundance of pathway “immune diseases” (*p* < 0.05); compared to the NAC group, the PEDV group increased the abundance of pathway “infectious diseases: parasitic” and decreased the abundance of pathway “excretory system” (*p* < 0.05). In the ileum, compared to the control group, the NAC group increased the abundance of pathways “signaling molecules and interaction” and “cellular community—eukaryotes” (*p* < 0.05); compared to the NAC group, the PEDV+NAC group decreased the abundance of pathways “cell motility,” “infectious diseases: bacterial” and “excretory system” (*p* < 0.05). In the colon, compared to the control and the NAC groups, the PEDV and the PEDV+NAC groups increased the abundance of pathways “cell motility,” “infectious diseases: viral,” and “circulatory system” (*p* < 0.05). In the cecum, compared to the control group, the PEDV group also increased the abundance of pathways “xenobiotics biodegradation and metabolism,” “cellular community—prokaryotes,” “cellular community—eukaryotes,” and “cancers: specific types” (*p* < 0.05); compared to the control and the NAC groups, the PEDV group increased the abundance of pathways “signal transduction,” “membrane transport,” “signaling molecules and interaction,” “cell motility,” “infectious diseases: bacterial,” “infectious diseases: viral,” “environmental adaptation,” and “circulatory system” (*p* < 0.05); compared the PEDV group, the PEDV+NAC group decreased the abundance of pathways “amino acid metabolism,” “signaling molecules and interaction,” “cellular community—eukaryotes,” “cancer—overview,” “drug resistance—antineoplastic,” “endocrine and metabolic diseases,” “cancers: specific types,” “endocrine system”, and “excretory system” (*p* < 0.05).

## Discussion

Several studies have revealed the dysbiosis of porcine gut microbiome induced by PEDV infection (13–17), which was associated with the increased abundance of pathogenic bacteria (e.g., *Shigella, Enterococcus*, and *Fusobacterium*) and the decreased abundance of short-chain fatty acid (SCFA)-producing bacteria (e.g., *Bacteroides, Prevotella, Butyricimonas*, and *Alistipes*). However, for the first time this study investigated the effect of a potential anti-PEDV substance on the gut microbiota in piglets, NAC was supplemented to the diet of both the healthy and PEDV-infected piglets. Our previous study indicated that NAC improved mucosal structure and intestinal permeability, reduced oxidative stress, and modulated the expression of genes associated with intestinal immune in the piglets during PEDV infection (25). Therefore, we hypothesize that the supplementation of NAC is promising to protect piglets from the microbiome dysbiosis caused by PEDV infection.

In the present study, the ileal samples displayed a higher similarity of bacterial community than other intestinal sections, as evaluated by insignificant data of alpha and beta diversity. However, several significant differences of diversity indices were observed in the jejunum, colon, and cecum, particularly the obvious clusters of beta diversity between the healthy and the PEDV-infected piglets, indicating the community of the gut microbiota was markedly altered by PEDV infection. Similar findings were obtained by Huang et al. (16) and (17). Interestingly, increased alpha diversity indices (Shannon and Simpson) were observed in the PEDV-infected piglets, implying PEDV infection led to the dysbiosis of porcine microbiome. This result is similar to the finding of Huang et al. (16) that alpha diversity increased with time during PEDV infection, but is contrary to results of Song et al. (12) and (17). Nevertheless, Chao index in the ileum was found as the only difference between the control and NAC groups, no other variation was found between the control and NAC groups and between the PEDV and the PEDV+NAC groups, suggesting a slight effect of NAC on the bacterial community.

Characterization of bacterial composition in this research demonstrated remarkable variations caused by PEDV infection. Profound shifts in the microbiota composition at the phylum level were found in the jejunum, colon, and cecum. Particularly, the PEDV and the PEDV+NAC groups displayed a lower abundance of *Bacteroidetes* and a higher abundance of *Proteobacteria, Firmicutes*, and *Fusobacteria* than the control and the NAC groups in the colon and cecum. The phylum *Bacteroides* is important for carbohydrate fermentation and polysaccharide catabolism, as well as amino acid and protein utilization (27). The phylum *Firmicutes* has been shown to be involved in energy resorption, and potentially related to the development of obesity and inflammation (28). Many studies suggested that *Proteobacteria* and *Fusobacteria* are associated with the imbalance of microbiota, inflammations, and various clinical anaerobic infections (29, 30). These results suggest that PEDV infection caused severe alterations in the composition of the gut microbiota in piglets, which could lead to the dysbiosis and affect intestinal metabolism and host health. However, similar microbiota composition was observed between the control and the NAC groups as well as between the PEDV and the PEDV+NAC groups, implying a limited effect of NAC supplementation on the composition of bacterial community in either the healthy or the PEDV-infected piglets.

The abundance of several OTUs showed significant differences between the healthy and the PEDV infected piglets. Interestingly, PEDV infection was associated with decreased proportions of *Shigella* and increased proportions of *Lactobacillus, Odoribacter, Anaerovibrio, Helicobacter, Lachnospiraceae*_g and *Sutterella*. *Shigella* species generally invade the epithelial lining of the colon, causing severe inflammation and death of the cells lining the colon, which could result in the diarrhea and even dysentery (31, 32). However, the reduction of *Shigella* in this study was only observed in the jejunum, which may be ascribed to the frequent flow of intestinal content induced by the diarrhea. Genera *Helicobacter* and *Sutterella* are typical pathogenic bacteria, which have been frequently associated with human diseases, such as stomach cancer and inflammatory bowel disease (33, 34). Increased *Helicobacter* and *Sutterella* abundances suggested the adverse healthy effect of PEDV infection. *Lactobacillus* is commonly investigated as probiotic agents, which influences host immunity and disease susceptibility (35). Increased *Lactobacillus* was also observed in PEDV-infected piglets (16), which was hypothesized to be the effect of pathogen inhibition or immunomodulation of the host. The genus *Odoribacter* and the family *Lachnospiraceae* are SCFAs-producing bacteria (36, 37), *Anaerovibrio* is often associated with lipolysis (38). The increase of these metabolic-related bacteria may involve in metabolic regulations of the host, or metabolic disorder induced by the dysbiosis during PEDV infection. Future investigation is warranted to unravel the complex relationship between the microbiome shifts and PEDV infection.

In this study, NAC supplementation presented different trends in the alteration of gut microbiome in the healthy piglets vs. the PEDV-infected piglets. In the healthy piglets, NAC supplementation decreased the proportion of family *Rikenellaceae* and increased the proportion of genera *Streptococcus* and *Enterococcus*; while in the PEDV-infected piglets, NAC was associated with decreased proportions of *Oscillospira* and *Prevotella*. Previous evidence showed that the members of family *Rikenellaceae* showed a high potential for fermenting dietary proteins (39), and enriched in mice fed a high-fat diet (40). Streptococcus populations are primary fermenters of diet-derived simple sugars in the small intestine (41), as evaluated by the high expression of genes involved in primary carbohydrate transport systems by the small intestinal *Streptococcus* (42). Decreased *Rikenellaceae* and increased Streptococcus by NAC supplementation indicated that NAC played a positive role in the metabolic regulation of the healthy piglets. Moreover, *Enterococcus* spp. have been reported to produce bacteriocins which inhibit Gram-positive food-borne bacteria and intestinal pathogens (43, 44), increased *Enterococcus* by NAC supplementation implied a potential to improve immunity of the healthy piglets. Recent research reported that *Oscillospira* is often associated with leanness (45), while *Prevotella* was negatively associated with the severity of diarrhea-predominant irritable bowel syndrome (46, 47). NAC supplementation decreased the abundance of *Oscillospira* and *Prevotella* in the PEDV-infected piglets, implying that NAC could promote the growth of piglets and mitigate diarrhea during PEDV infection. Additionally, NAC supplementation increased the abundance of *Lactobacillus* in both the healthy and the PEDV-infected piglets, suggesting a beneficial effect of NAC on the regulation of the gut microbiota in the suckling piglets.

A few studies have revealed changes in the functional potential of microbiome in relation to PEDV infection, which was found to attenuate metabolism and defense mechanisms and enhance transcription and membrane transport (15–17). Similar results were obtained in the present study, reflecting that attenuating metabolism, barrier, and immune is the major functional shift of microbiome during PEDV infection. However, in the PEDV-infected piglets, many functional potentials of the microbiome were remarkably influenced by NAC supplementation. For instance, decreased amino acid metabolism, cell signaling, cellular community, multiple disease-related pathways, and endocrine and excretory system in the cecum. These results demonstrated that functional changes of the gut microbiome may be a way that NAC protects the piglets from PEDV infection.

## Conclusion

This study evaluated the effect of dietary supplementation with NAC on the gut microbiome in the healthy and PEDV-infected piglets. PEDV infection caused severe dysbiosis of gut microbiome, as demonstrated by the community variation (beta diversity) and the composition shifts (decreased *Shigella* and increased *Lactobacillus, Odoribacter, Anaerovibrio, Helicobacter, Lachnospiraceae*_g, and *Sutterella*), resulted in functional changes associated with metabolism, barrier and immune. However, NAC supplementation played a positive role in regulating the gut microbiome during PEDV infection, as indicated by the altered bacterial populations (decreased *Oscillospira* and *Prevotella* and increased *Lactobacillus*) and function potentials (amino acid metabolism, cell signaling, cellular community, diseases, endocrine, and excretory system). Therefore, regulating the gut microbiome by anti-PEDV(antiviral) substances or compounds could be taken as a promising candidate for the prevention or treatment of PEDV infection.

## Data Availability Statement

The original contributions presented in the study are deposited in the Majorbio Cloud Platform database (www.majorbio.com). The datasets generated for this study are available on request to the corresponding author.

## Ethics Statement

The animal study was reviewed and approved by Animal Care and Use Committee of Wuhan Polytechnic University.

## Author Contributions

TW and YH planned the project and designed the experiments. TW, YL, and XL conducted the experiments. YL carried out the data analysis. MW, KY, SL, and CJ contributed the animal trials. XL and DZ contributed reagents preparation and samples collection. YL and TW wrote the manuscript and reviewed by QZ, YZ, and DY. All authors have read and agreed to the published version of the manuscript.

## Conflict of Interest

The authors declare that the research was conducted in the absence of any commercial or financial relationships that could be construed as a potential conflict of interest.
